# Longevity Estimates of Canary Palms and Dragon Trees via Radiocarbon Dating: Initial Results

**DOI:** 10.3390/plants13010045

**Published:** 2023-12-22

**Authors:** Franco Biondi, Guaciara M. Santos, Priscila Rodríguez Rodríguez, Pedro A. Sosa

**Affiliations:** 1 DendroLab, Department of Natural Resources and Environmental Science, University of Nevada, Reno, NV 89557, USA; 2 Department of Earth System Science, University of California Irvine, Irvine, CA 92697, USA; gdossant@uci.edu; 3 Instituto Universitario de Estudios Ambientales y Recursos Naturales (IUNAT), Universidad de las Palmas de Gran Canaria, 35017 Las Palmas de Gran Canaria, Spain; priscila.rodriguez@ulpgc.es (P.R.R.); pedro.sosa@ulpgc.es (P.A.S.)

**Keywords:** radiocarbon, ^14^C dating, plant longevity, Canary Islands, *Phoenix canariensis*, *Dracaena draco*

## Abstract

Correctly estimating the maximum lifespan of plant species is a necessary component of demographic and life-history studies, which, in turn, are needed for understanding climatic impacts. Arboreal monocotyledons, which can grow to >30 m in height and >5 m in trunk perimeter, are difficult to age because they do not undergo seasonal dormancy; hence, their longevity has been estimated using various size-related methods. In this study, we tested radiocarbon (^14^C) dating with Accelerator Mass Spectrometry (AMS) as an additional tool for determining the age of two iconic monocotyledons: the Canary Island palm and the dragon tree. A total of 25 samples were collected from the basal stem of four palms and five dragon trees on Gran Canaria and Tenerife and then processed using the most advanced ^14^C-AMS analysis available. Calibration curves provided by the “IntCal group” were used to determine the oldest possible age of each sample, and 16 of them were found to be “modern”, i.e., formed after the 1950s. Nine samples that were either collected from exterior, but lignified, palm tissues or from interior, and lignified, dragon tree tissues suggested ages > 300 years. Given the constant improvement of ^14^C-AMS tools, they can contribute to the further refinement of existing scientific knowledge on Macaronesian charismatic megaflora.

## 1. Introduction

Plant longevity is a key ecological trait that influences ecosystem structure, function and dynamics, including global carbon cycling [1]. For instance, information on tree maximum ages has recently challenged the long-held notion that shade-tolerant, late-successional species have longer lifespans than early-successional species by pointing out that the longest-living tree species do not fit this paradigm [2]. As longevity is complementary to mortality, and natural ecosystems worldwide have experienced die-offs linked to drought episodes [3], conservationists need to understand the life-history traits of plants to clarify climatic impacts and to develop effective management strategies, especially for threatened species that survive in rare habitats [4].

A basic limitation for determining plant ages is found in monocotyledons, which usually lack a secondary meristem for diameter growth [5,6] and therefore cannot be dated using the seasonal formation of xylem (or wood) layers [7]. Despite this feature, monocotyledon species can grow to be very tall (>30 m height), as palms notably do, or develop impressive trunks (>5 m perimeter), as in dragon trees [8]. The stem structure and hydraulic functioning of arboreal monocotyledons is far from simple and could be better understood once multiple stem sections, taken in sequence, were analyzed as cinematic sequences [6]. Briefly, palm stems are formed by a matrix of non-uniform parenchyma tissues that contain primary vascular bundles (xylem on the inside, phloem on the outside) linking roots to leaves. In many species, although the bundles are found throughout the stem, it is possible to distinguish an inner and an outer vascular system, which remain connected in complex ways and become increasingly lignified and mechanically stronger, mainly at the stem periphery, as the plant grows in height and age [9]. Basal stem enlargement, while only from primary developmental processes, may cease early or late in the plant’s life, resulting in more cylindrical or conical stem shapes, respectively [10]. In addition, a few monocotyledons (e.g., *Dracaena*) also develop a lateral meristem [8,11] in the outer system that produces secondary vascular bundles and tissues mostly towards the inside, allowing for diameter growth throughout their lifetime.

Because their stem cell types are physiologically active throughout the year, palms do not undergo dormancy, which therefore limits their geographical distribution to tropical regions and mild climates without freezing temperatures [10]. The Canary Islands, which are outside the tropics but where the cool trade winds and the cold surface waters create relatively temperate conditions [12], represent the natural habitat of two arboreal—and iconic—monocotyledons: the Canarian date palm (*Phoenix canariensis* H. Wildpret) and the dragon tree (*Dracaena draco* (L.) L.). The former species is one of the three autochthonous palm species within European territory, together with *Chamaerops humilis* L. and *Phoenix theophrasti* Greuter. *Dracaena draco* is endemic to the Macaronesian region (i.e., the archipelagos of Madeira, the Canary Islands and Cape Verde) and the Moroccan Anti-Atlas. In the Canary Islands it is found naturally only on the islands of Tenerife and Gran Canaria, and it is unclear why it does not occur on other islands such as La Gomera and La Palma because many aspects of its reproductive biology are unknown [13].

The unique endemic palm groves of the Canary Islands have been catalogued as priority habitats of the European Union Natura 2000 network of protection areas and are listed in the European Union Habitats Directive Annex I (Habitat Code 9370). While they are considered to have an ‘Unfavourable–Inadequate’ conservation status in the Macaronesian region [14], such natural groves constitute a representative and distinctive element of the Canarian vegetation landscape, contributing to its identity, culture, religion, environment and even its economy. This relevance has motivated the designation of *Phoenix canariensis* as the vegetal symbol of the Canary Islands by the Canarian Government [15].

The largest arboreal monocotyledons are extraordinary enough to attract not only tourists, but also the efforts of ecologists and conservationists, e.g., [16], and can then be characterized as charismatic megaflora, even though this definition has typically been reserved to trees and forests [17]. Because of both popular and scientific curiosity, the age of Canary palms and dragon trees has been investigated for some time using growth functions [18], i.e., by identifying statistical relationships between size features and plant age. The longevity of dragon trees is usually derived from the growth of new branch segments induced by flowering events [19,20], whereas the age of Canary palms has been inferred from the stipe/crown vertical length ratio [21].

Most of these statistical estimation models rely on correctly identifying past growth patterns from current external features as well as on linearity assumptions, which are often violated by age–size relationships, particularly in large/old individuals, e.g., [22], even for palms [9]. In addition, estimates from these models are often outside the range of ages and size parameters used for model fitting, which is a notoriously problematic issue [23]. It is then not surprising that, in their recent review of the scientific literature on dragon trees, [24] concluded that “we still do not understand how to estimate age reliably”. Despite such concerns and limitations, no radiocarbon dating study has yet targeted the arboreal monocotyledons of the Canary Islands.

Radiocarbon (^14^C) dating of tropical woods used to be considered unreliable for the period “between 1650 and 1940” [25], and therefore it has not been widely applied in tropical ecology; cost and analytical difficulty have played a role as well [26]. In reality, during the past quarter of a century, AMS (Accelerator Mass Spectrometry) has made ^14^C dating much faster and cheaper, and only ~30 mg of tissue (about the average weight of a grain of rice) are needed for each sample [27]. The calibration of radiocarbon dates led by the international IntCal Working Group (https://intcal.org/, accessed on 15 December 2023) has also made progress, and research on that subject continues at a fast pace. Because radiocarbon calibration curves are not smooth and monotonic, they cannot be mathematically inverted and therefore require statistical methods to obtain the most reliable dates [28].

While recent ^14^C calibration curves still contain wiggles and plateaus, several sections have been improved using single-year tree-ring measurements [29]. These measurements relate to abrupt ^14^C excursions first detected in Japanese cedar from 774–775 CE due to extraterrestrial events [30]. Other rapid ^14^C changes have been since identified in tree rings, including 993–994 CE [31], ~660 BCE [32], 3372 BCE [33] and 5480 BCE [34]. Thus, annually resolved ^14^C measurements on different wood subsets have been produced by various laboratories in order to improve the resolution of the ^14^C calibration curve and then added to IntCal data [29,35]. In turn, such improvements have allowed for calendar age calibration refinements (e.g., [36]).

In a modern world characterized by the constantly increasing speed of technological changes, monumental plants of impressive size remind us of the everlasting power of nature [37]. They are silent witnesses—symbols of steadfast endurance to disturbances both environmental and human. As they remain visible in the landscape throughout multiple human generations, they become intimately linked with socio-economic structures, traditional customs and spiritual beliefs, to the point that their very presence becomes a synonym of sustainable land stewardship [38]. It is therefore not surprising that local communities advertise their presence and often embellish their value by exaggerating their ages.

Claims of multi-millennia-old plants are now easily found on the internet and are given credit even by supposedly reputable sources. As an example, the monumental olive tree (*Olea europaea* L.) located in the small town of Ano Vouves on the island of Crete is claimed to be 2000–4000 years old [39,40]. Olives have been a fundamental ingredient of Mediterranean diets for millennia, and the trees that produce them can indeed live for a long time, but in reality olive wood is notoriously difficult to examine dendrochronologically [41], and even the oldest radiocarbon-dated olive stems do not reach a single millennium [42]. Our study had therefore two main objectives: (a) to test radiocarbon dating as an additional tool to estimate the longevity of arboreal monocotyledons and (b) to determine field sampling procedures and laboratory methods most appropriate for the task.

## 2. Results

**Figure 1:**
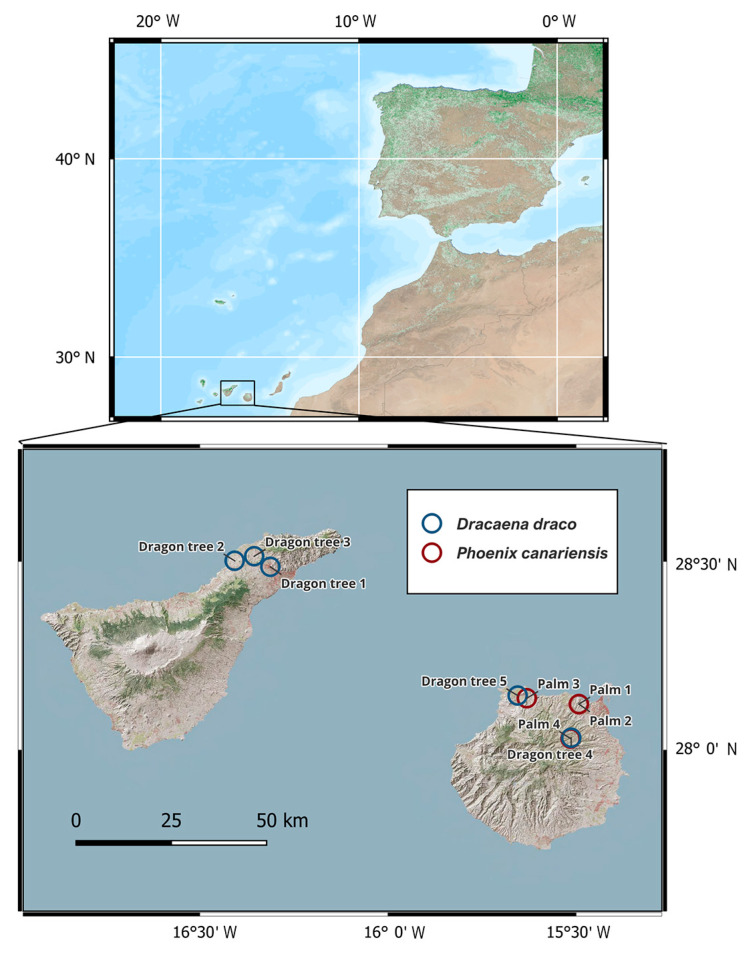
Map of sample locations in the Canary Islands (top panel), with palms (*Phoenix canariensis*) on Gran Canaria and dragon trees (*Dracaena draco*) on both Gran Canaria and Tenerife (Table 1; because of their proximity, the blue circle for “Palm 4” is obscured by the red circle for “Dragon tree 4”).

Permission was granted by local landowners and managers to sample four palms and five dragon trees, all of monumental size (Table 1), on the islands of Gran Canaria and Tenerife (Figure 1). The 25 tissue samples collected in the field consisted of enough material to produce graphites in the laboratory for high-precision ^14^C analysis (Table 2). While typical recovery yields after cellulose extractions are ~30% from present-day tropical and subtropical trees and ~10% from ancient woods and barley [27], recovery was ~8–9% for several samples (Table 2), even without including the hemicellulose removal step of 17% NaOH that is included in the alpha-cellulose procedure. Samples were followed by reference materials, whose F^14^C values averaged 1.1027 ± 0.0001 (*n* = 2) for FIRI-J barley and 0.0024 ± 0.0008 (*n* = 2) for the AVR wood blank, which were both within expected values [27].

Radiocarbon calibration curves provided by the IntCal group (i.e., IntCal20 [29] and Northern Hemisphere Zone 2 [43]) were used to determine the oldest age of each sample, and 16 of them were found to be “modern” (Table 2), which means they were formed after the mid-20th century thermonuclear weapon tests, or more specifically after 1950 CE [44]. These tissues were obtained from relatively soft areas of palm basal stems or from the outside of dragon trees because no hard surfaces were exposed (in palms) or no stem cavities were present (in dragon trees).

**Table 1:** Summary information on plants that were sampled for radiocarbon dating in the Canary Islands. Palm = *Phoenix canariensis* H. Wildpret; Dragon tree = *Dracaena draco* (L.) L.

Plant	No. of Samples	Sampling Date	Height (m)	Perimeter (m)	Estimated Age (yrs)	Latitude °N	Longitude °W	Location
Palm 1	2	4 April 2023	36 ^6^	2.8 ^6^	335 ^1^	28.12183	15.49251	Tenoya, Gran Canaria
Palm 2	2	4 April 2023	27 ^6^	2.5 ^7^	312 ^1^	28.12183	15.49251	Tenoya, Gran Canaria
Palm 3	2	4 April 2023	10 ^7^	1.6 ^7^	220 ^2^	28.13752	15.63104	Guía, Gran Canaria
Palm 4	2	5 May 2023	15 ^7^	1.7 ^7^	280 ^2^	28.03045	15.51475	Santa Brígida, Gran Canaria
Dragon tree 1	3	4 May 2023	5 ^8^	5 ^8^	300 ^3^	28.48587	16.31256	La Laguna, Tenerife
Dragon tree 2	4	4 May 2023	12 ^9^	6.6 ^9^	345 ^3^	28.50191	16.40748	Tacoronte, Tenerife
Dragon tree 3	4	4 May 2023	8 ^10^	6.9 ^10^	345 ^3^	28.51399	16.35552	Tegueste, Tenerife
Dragon tree 4	4	5 May 2023	17 ^4^	4.8 ^4^	230 ^4^	28.03255	15.51308	Santa Brígida, Gran Canaria
Dragon tree 5	2	5 May 2023	8 ^4^	4 ^5^	300 ^5^	28.14471	15.65532	Gáldar, Gran Canaria

^1^ From Table 5 in [21]. ^2^ Estimated using the method proposed by [21]. ^3^ From [45]. ^4^ From Table 2 in [46]. ^5^ Reported in [47]. ^6^ Reported in [48]. ^7^ Estimated from photographs. ^8^ Reported in [49]. ^9^ Reported in [50]. ^10^ Reported in [51].

**Table 2:** Summary information on radiocarbon (^14^C-AMS) analysis of plants listed in Table 1.

Plant	Weight (mg)	Holocellulose (mg)	Recovery Yield (%) *	Fraction Modern (F^14^C ± 1σ)	^14^C Calibrated (yr CE) **	Field Notes
Palm 1	21.7	2.0	9.2	1.0526 ± 0.0017	1957.16 ± 0.14	Soft exterior
	29.6	2.1	7.1	1.0602 ± 0.0018	1957.36 ± 0.14	Soft exterior
Palm 2	26.2	2.3	8.8	1.0211 ± 0.0019	1956.11 ± 0.37	Soft exterior
	24.2	2.1	8.7	1.0285 ± 0.0020	1956.43 ± 0.27	Soft exterior
Palm 3	23.7	1.4	5.9	1.1438 ± 0.0021	1958.73 ± 0.08	Soft exterior
	28.6	1.7	5.9	1.0249 ± 0.0017	1956.29 ± 0.25	Soft exterior
Palm 4	30.8	10.6	34.4	0.9670 ± 0.0017	1539 ± 12	Hard interior
	30.9	3.4	11.0	0.9750 ± 0.0020	1669 ± 14	Hard interior
Dragon tree 1	35.9	2.7	7.5	1.5007 ± 0.0021	1963.17 ± 0.05	Exterior
	28	8.1	28.9	1.0725 ± 0.0019	1957.70 ± 0.17	Exterior
	38.6	12.1	31.3	1.0869 ± 0.0018	1958.00 ± 0.13	Exterior
Dragon tree 2	31.6	4.4	13.9	1.0082 ± 0.0018	1955.59 ± 0.40	Exterior
	35	2.6	7.4	0.9770 ± 0.0016	1674 ± 11	Interior
	34.5	12.8	37.1	0.9862 ± 0.0018	1709 ± 18	Fallen branch
	30.3	6.5	21.5	0.9755 ± 0.0018	1671 ± 13	Interior
Dragon tree 3	35.6	8.2	23.0	0.9765 ± 0.0019	1673 ± 12	Hard interior
	37.7	8.8	23.3	0.9694 ± 0.0018	1652 ± 12	Exterior high
	28.7	3	10.5	0.9949 ± 0.0017	1714 ± 5	Hard interior
	37	10	27.0	1.0352 ± 0.0018	1956.64 ± 0.25	Exterior
Dragon tree 4	36.1	4.3	11.9	1.0354 ± 0.0015	1956.64 ± 0.24	Exterior
	34.3	1.7	5.0	1.0777 ± 0.0018	1957.81 ± 0.11	Exterior
	29.1	5	17.2	1.0390 ± 0.0018	1956.79 ± 0.15	Exterior
	25.6	1.9	7.4	1.0105 ± 0.0018	1955.68 ± 0.39	Exterior
Dragon tree 5	29.8	2.1	7.0	1.4749 ± 0.0019	1963.06 ± 0.07	Exterior
	33.5	9.9	29.6	0.9814 ± 0.0016	1684 ± 13	Exterior

* Percentage of “Holocellulose (mg)” obtained from the original sample “Weight (mg)”. ** Oldest age ranges derived from calibration curves using 95% confidence intervals.

**Figure 2:**
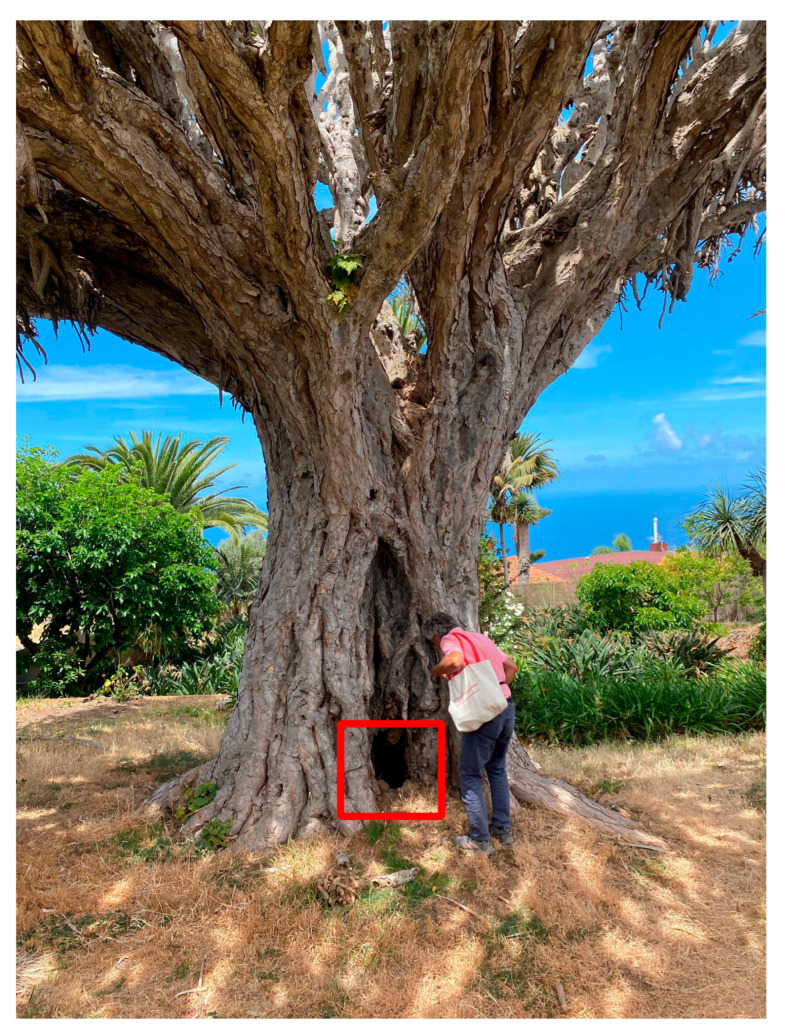
A dragon tree sampled on Tenerife (“Dragon tree 2” in Table 1 and Table 2) whose radiocarbon dates could be based on tissues taken from inside the stem because of a large cavity (red rectangle; see also Figure S1), which is being inspected by the first author.

Nine samples collected either from interior, lignified, dragon tree tissues (e.g., Figure 2 and Figure S1) or from exterior, but lignified, palm tissues (e.g., Figure S2), revealed stem ages of 300 years or more (Table 2). The two samples with ages >300 years that were taken from the outside of dragon trees 3 and 5 (Table 2) came from stem areas that had stopped growing in the past (Figure S3, bottom). The oldest calibrated age of 1671–1697 CE was assigned to the last sample in Table 2 because of our methodology, which always selected the oldest age ranges yielded by our very precise (i.e., with narrow errors) ^14^C results.

## 3. Discussion

Two of the most iconic plant species found in the Macaronesian islands of the Atlantic Ocean are the dragon tree and the Canary palm. Their arborescent, pachycaulous growth form has made them a regional symbol, even though both species are now found across the globe due to human cultivation. In terms of their conservation status, the International Union for Conservation of Nature and Natural Resources (IUCN) places *Phoenix canariensis* in the ‘Least Concern’ category [52] while *Dracaena draco* is considered ‘Endangered’ [53]. The latter species is also considered an example of the “randflora” biogeographic pattern, where the Germanic word “rand” stands for edge or border, and the term indicates unrelated plant lineages with comparable disjoint distributions occurring throughout the continental margins of Africa and neighboring oceanic archipelagos, forming ‘a ring and leaving the center of the continent hollow’ [54]. Climate-driven vicariance, which can force species to migrate or persist only in residual areas, followed by long-distance dispersal, is usually considered responsible for randflora patterns [55]. Given the uncertainty that surrounds the ecology of these plant species, including their longevity, it is worth exploring additional scientific tools for determining their maximum ages.

Overall, our field sampling was successful only in a few cases, as most samples (6 out of 8 for palms, and 10 out of 17 for dragon trees) were dated post-1950s and not representative of plant longevity. While we were limited in our sampling strategy by the need to avoid stem damage, it is possible that “modern” samples could have contained recently formed tissues. Since holocellulose extractions remove non-structural carbon from the organic material to be dated, including water and sap intrusions, ^14^C-AMS results for our sample tissues suggest, at least for palms, the formation of new cells. It should be noted that palms maintain the physiological capability to form adventitious roots from the basal stem during their lifetime, but how these new tissues are formed is unclear [10]. Since the initiation zone of aerial roots can extend more than 1–2 m up the trunk in *Phoenix* sp., palm tissues that were dated as “modern” could have been affected by this peculiar growth feature. In fact, our purpose was instead to obtain tissues—even towards the outside of the stem—that represented plant longevity because, in palms, the xylem, phloem and even parenchyma cells can remain alive for the life of the plant [10].

With regard to “modern” samples from dragon trees, they were most likely related to either dead branches or recent extensions of the trunk. While ^14^C measurements on olive trees [56] and African baobabs [57] have shown that those woody species do not necessarily maintain growth over the stem perimeter, *Dracaena*-type wood is produced towards the inside by a lateral meristem; hence, the innermost tissues are assumed to be the oldest [8,11,58]. Since two samples collected from the outside of dragon trees (“Exterior” in Table 2) suggested ages >300 years, it is, however, plausible that certain parts of the stem stop growing, either because of damage to the lateral meristem or for yet unknown physiological reasons. We should also note that another possible age range for the pre-modern “Dragon tree 5” specimen was 1723–1767 CE, which is in better agreement with the age of “195–210” years that was reported in 2003 [46]. The “Drago del Ayuntamiento de Gáldar” [47] is in fact one of the best-known monumental plants on the island of Gran Canaria, being located in the patio of the old town hall building (Figure S3, top). An age of at least 300 years has been repeatedly attributed to this dragon tree based on a 1718 planting date, against which [46] convincingly argued using both historical documents and size features.

The presence of moisture, either from water or sap, in field samples did not affect the ^14^C-AMS dates because of the chemical treatments that were applied in the laboratory. Palms do store large quantities of water in their trunks, probably within the vacuoles of living cells [59,60]. According to [10], “That the water is not stored in intercellular spaces is demonstrated by cutting into the trunk. Water does not flow freely when an incision is made.” Dragon trees, given the xeric environments where they live, should also store abundant water in their stem, as we also observed when cutting a section from a massive branch that had previously fallen off “Dragon tree 2” (Figure S4a). The branch interior was still quite moist and even allowed fungi to grow in it (Figure S4b). The radial piece that was obtained from the branch section, when it was left out to dry, developed the same fungus (Figure S4c), suggesting that a large amount of moisture was initially retained in it.

Although the “modern” dates we obtained do not provide information on plant longevity, they provided useful, albeit negative, information on sampling strategy. The same could be said for the striking differences found in terms of recovery yields, which for our tissues were ~1/3 of modern tropical wood. These results are central for designing further research on monocotyledon longevity using radiocarbon dating. While age estimates from growth functions are commonly used, the scientific determination of plant maximum lifespan, when dendrochronological methods are not suitable, requires radiocarbon dating [42].

Radiocarbon in the atmosphere changed from about 1650 to 1950 CE in such a way that approximately the same result is obtained for a radiocarbon date over that entire age range. Rather than a radiocarbon dating problem, this issue is related to upper atmosphere ^14^C formation together with the rate of radiocarbon decay: when both parameters match for long periods of time, a plateau appears in the ^14^C calibration curve, and precise radiocarbon measurements result in extended calendar age ranges [61]. In this study, we relied on available information for the sampled plants to infer that the oldest ages should be reported.

While Canary palms and dragon trees have not been the target of radiocarbon dating until now, at least two other palm species have been investigated using radiocarbon methods [62,63]. In those studies, emphasis was placed on ^14^C concentrations related to the “bomb spike” of the early 1960s, which is a useful marker often employed even for verifying uncertain tree-ring dating [64]. Furthermore, tissue samples were pre-treated with acid–base–acid washes only, and, while chemical methods based on extracting holocellulose from wood are sufficient to remove most if not all non-structural compounds [27], acid–base–acid processing normally fails to remove contaminants with aromatic rings [65].

In conclusion, we found that, in order to obtain reliable radiocarbon dates from Canary palms and dragon trees, only lignified tissues ought to be sampled. The amount of moisture in the samples does not matter, because of the way samples are pretreated. Given their low recovery yields, it is also necessary to avoid the alpha-cellulose step during laboratory processing. It is possible that ^14^C-AMS calibration will improve in the near future, as ongoing research by the IntCal group is addressing the three-century ^14^C plateau that occurs just before 1950 [66]. Our own current and future research efforts are aimed at collecting additional samples, especially from a single plant in a sequence, as an attempt at ^14^C wiggle-match dating, where calibration is facilitated by having radiocarbon dates of multiple samples separated by a known, or even approximated, amount of time [28,67].

Besides their botanical and ecological value, the Canary Islands palm and dragon tree have acquired numerous social and cultural meanings, including traditional and medicinal significance related, for example, to the valuable crimson red resin known as “dragon’s blood” [68]. Particularly famous is the “Drago de Icod de los Vinos”, whose basal perimeter is ~20 m and is one of the symbols of Tenerife, not just for its impressive size but also for its historical significance and for the aesthetic experience it generates [69]. It was declared a “Monumento Natural de Interés Nacional” in 1917, and is popularly considered to be a thousand years old, although it is most likely less than half that age [24]. Because of the need to clarify the maximum lifespan of these unique species, our initial ^14^C-AMS dating study represents a contribution to further refine existing scientific knowledge on Macaronesian charismatic megaflora.

## 4. Materials and Methods

Field sampling was performed during April and May 2023 by taking wood samples from the stem of four Canary palms and five dragon trees (Table 1). Samples were usually collected near the trunk base and from areas that appeared hardened and oldest. Because these large, old plants often have irregular shapes near the ground, we could not always sample at the same height. Since we were not allowed to use an increment borer, most samples came from the outside of the stem after shaving a few mm off the surface. We then made an equally small incision to carve away the tissue sample. Stem surface scraping and sample carving were both performed by hand using a steel wood chisel with a ~5-mm tip width. Subsequently, with tweezers, we took small pieces of tissue that was not previously exposed to the outside. While we aimed for hard (lignified) tissues, the incision occasionally revealed that underneath the surface there were still soft and moist tissues. A few samples could be taken from the stem interior, either because of the cavities that dragon trees typically develop near the ground as they grow older and radially larger (Figure 2 and Figure S1) or because of partial stem damage that had eroded away the outside of a palm trunk (Figure S2).

Care was taken to minimize contamination of the samples during extraction, storage and transport to the laboratory. At the University of California-Irvine KCCAMS laboratory, during chemical processing, alpha-cellulose treatment was avoided because of cross-linked fiber structures in most samples. Thus, holocellulose extraction and homogenization were performed together with 5–6 baths/rinses of 1N NaOH to remove soluble compounds [27] prior to combustion and graphitization [70].

As expected, statistical errors associated with ^14^C data increased slightly after isotopic fractionation corrections using δ^13^C values measured on prepared graphite using the AMS spectrometer and after the subtraction of blanks for background corrections. Blanks (^14^C-free wood, or AVR) and post-bomb reference material (FIRI-J barley mash) were chemically processed alongside the samples and together with modern tropical wood. The following combustible reference materials were measured to standardize values and to check measurement accuracy: six aliquots of HOx1 (or NIST HOxI SRM 4990B) and one aliquot each of HOx2 (or NIST HOx2 SRM-4990C) and ANU (sucrose).

Recovery yields were considerably lower than those from most tropical woods. Radiocarbon concentrations were given as fractions of the modern standard (F^14^C) and as conventional radiocarbon ages [71]. Calendar ages were obtained from the online CALIBomb tool [35], which derives both pre- and post-bomb calibrated calendar years using the most updated datasets. We used the “IntCal20” curve for results before 1950, or pre-bomb [29], and the “NHZ2” (Northern Hemisphere Zone 2) calibration curve for post-bomb results, as it is most appropriate for samples from approximately ~40° N to the latitude of the mean summer intertropical convergence zone after 1950 [43,72].

## Data Availability

The data presented in this study are contained within the article or its Supplementary Materials.

## Supplementary Materials

The following supporting information can be downloaded at: https://www.mdpi.com/article/10.3390/plants13010045/s1, Figure S1: The basal cavity of the dragon tree shown in Figure 2; Figure S2: A Canary palm sampled on Gran Canaria (“Palm 4” in Table 1 and Table 2); Figure S3: Photographs of “Drago del Ayuntamiento de Gáldar”, which is “Dragon tree 5” in Table 1 and Table 2; Figure S4: The branch section from “Dragon tree 2” revealed a large amount of moisture stored inside the stem.
