# Supplementary material for: Longevity Estimates of Canary Palms and Dragon Trees via Radiocarbon Dating: Initial Results
Source: Plants (Basel). 2023 Dec 22;13(1):45. doi: 10.3390/plants13010045 (PMC10780642; doi:10.3390/plants13010045)
Supplement: Supplementary file 1 [file plants-13-00045-s001.zip › plants-2771618-supplementary.pdf]

Supplementary Materials for

Longevity estimates of Canary palms and dragon trees via radiocarbon dating: Initial results

Franco Biondi [1,*], Guaciara M. Santos [2], Priscila Rodríguez Rodríguez [3] and Pedro A. Sosa [3]

[1] DendroLab, Department of Natural Resources and Environmental Science, University of Nevada, Reno, NV 89557, USA; franco.biondi@gmail.com
[2] Department of Earth System Science, University of California Irvine, Irvine, CA, USA; gdossant@uci.edu
[3] Instituto Universitario de Estudios Ambientales y Recursos Naturales (IUNAT), Universidad de las Palmas de Gran Canaria, Canary Islands, Spain; priscila.rodriguez@ulpgc.es (P.R.R.); pedro.sosa@ulpgc.es (P.A.S.)

* Correspondence: franco.biondi@gmail.com; Tel.: +1 775 784-6921

This file includes:

Figure S1: The basal cavity of the dragon tree shown in Figure 2, which allowed for collecting samples from inside the stem (red circle shows the location of "Interior" sample of "Dragon tree 2" in Table 3).

Figure S2: A Canary palm sampled on Gran Canaria ("Palm 4" in Tables 1 and 2) whose radiocarbon dates could be based on hard tissues taken from inside the stem because of basal damage. **Left**: the entire palm. **Top right**: the basal portion, with a damaged area that is being inspected by the last author. **Bottom right**: Location of the first sample taken from the "Hard interior" (Table 2), with tweezers placed just below it (red ellipse) for scale.

Figure S3: Photographs[1] of "Drago del Ayuntamiento de Gáldar", which is "Dragon tree 5" in Tables 1 and 2. This example of charismatic megaflora is located in the patio of the old town hall building (**top**), and was sampled by the first and the last author in an area of the stem that had stopped growing in the past (**bottom**), resulting in a > 200-year radiocarbon date.

Figure S4: The branch section from "Dragon tree 2" revealed a large amount of moisture stored inside the stem. **(a)**: Cutting the cross-section from the branch. **(b)** The section (a blue pen was placed on it for scale) revealed a round-shaped cavity inside the stem where fungi (red circle) could grow. **(c)** A radial piece that was cut from the branch section (left photograph), once left out on a bench to dry, developed the same white fungi (right photograph) that had been observed in the field.

[1] Both photographs are publicly available on the Facebook page maintained by the local administrators (https://www.facebook.com/dragodegaldar/).

Figure S1. The basal cavity of the dragon tree shown in Figure 2, which allowed for collecting samples from inside the stem (red circle highlights the location of the first “Interior” sample from “Dragon tree 2” in Table 2).

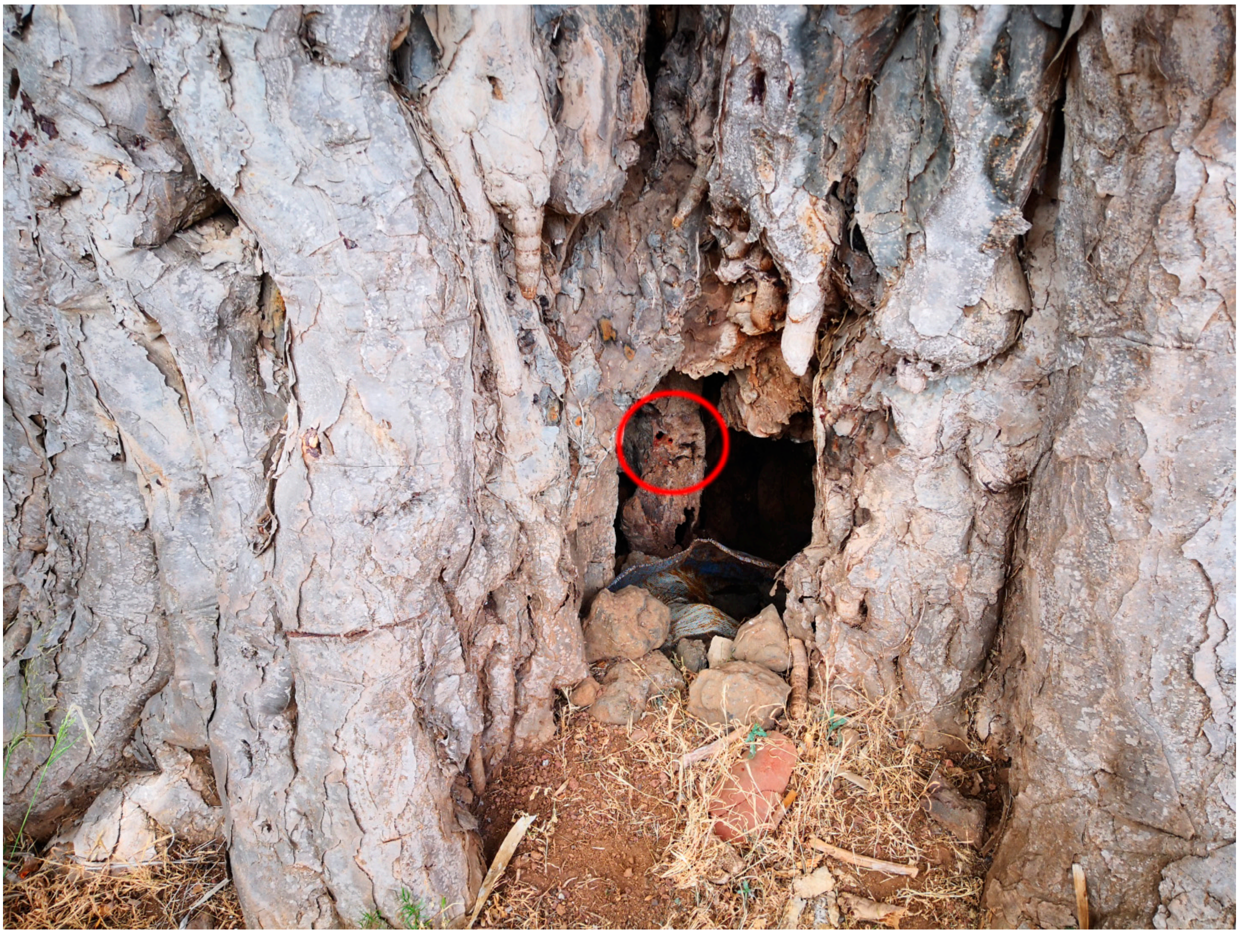

Figure S2. A Canary palm sampled on Gran Canaria ("Palm 4" in Tables 1 and 2) whose radiocarbon dates could be based on hard tissues taken from inside the stem because of basal damage.

Left: the entire palm. **Top right**: the basal portion, with a damaged area that is being inspected by the last author. **Bottom right**: Location of the first sample taken from the "Hard interior" (Table 2), with tweezers placed just below it (red ellipse) for scale.

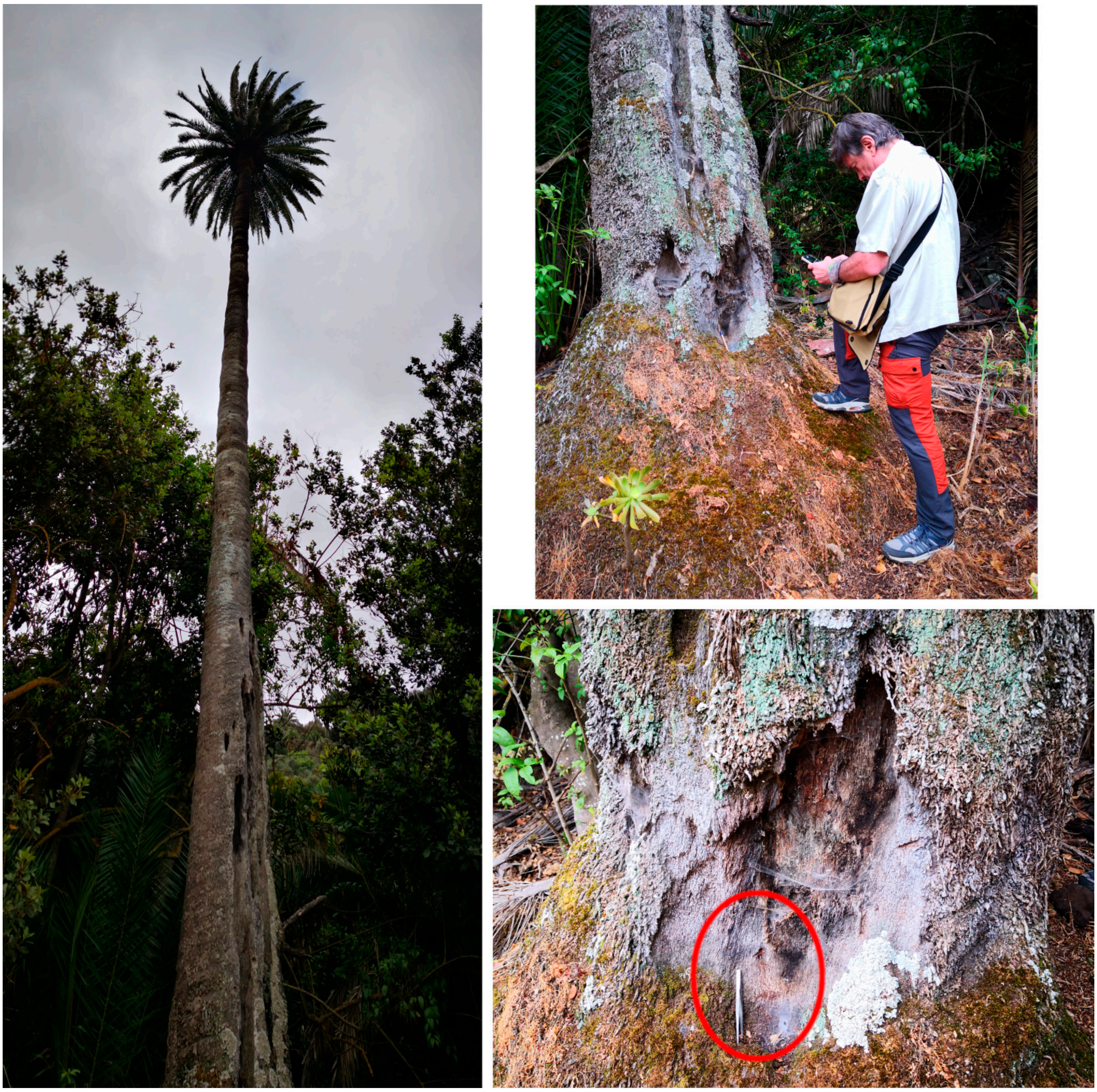

Figure S3: Photographs[2] of "Drago del Ayuntamiento de Gáldar", which is "Dragon tree 5" in Tables 1 and 2. This example of charismatic megaflora is located in the patio of the old town hall building (**top**), and was sampled by the first and the last author in an area of the stem that had stopped growing in the past (**bottom**), resulting in a > 200-year radiocarbon date.

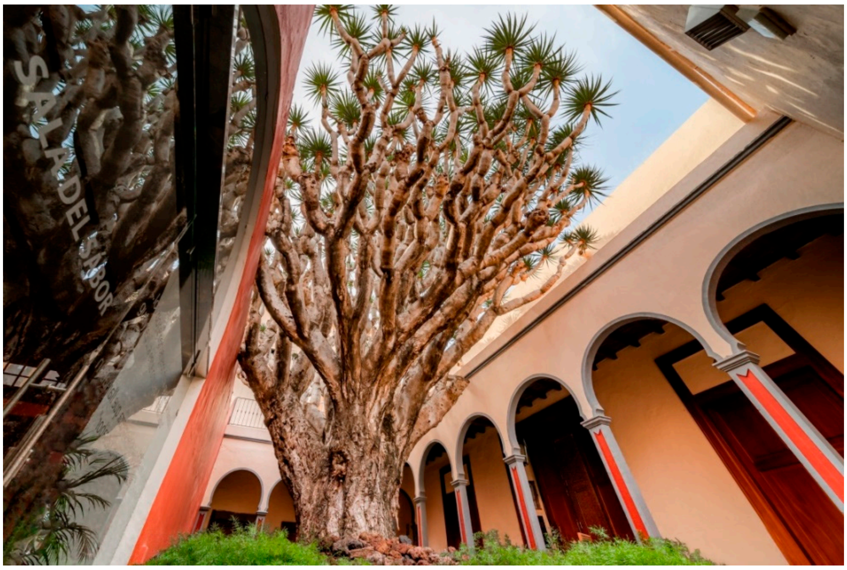

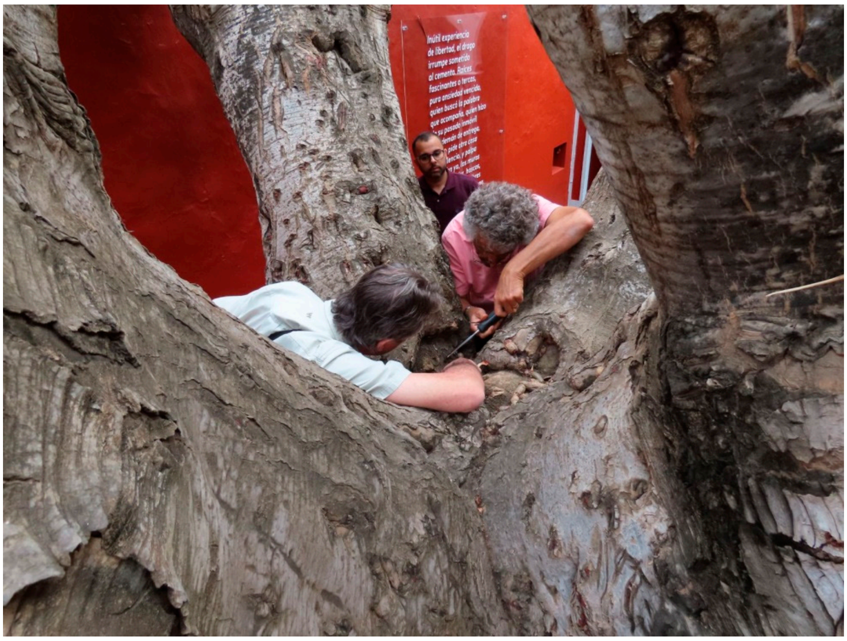

[2] Both photographs are publicly available on the Facebook page maintained by the local administrators (https://www.facebook.com/dragodegaldar/).

Figure S4: The branch section from "Dragon tree 2" revealed a large amount of moisture stored inside the stem. **(a)** Cutting the cross-section from the branch. **(b)** The section (a blue pen was placed on it for scale) revealed a round-shaped cavity inside the stem where fungi (red circle) could grow. **(c)** A radial piece that was cut from the branch section (left photograph), once left out on a bench to dry, developed the same white fungi (right photograph) that had been observed in the field.

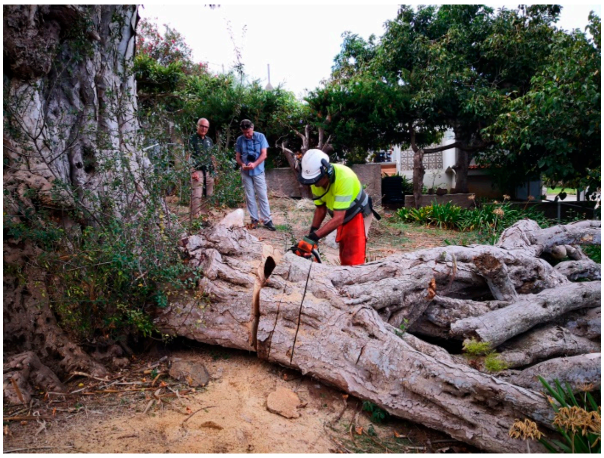

(a)

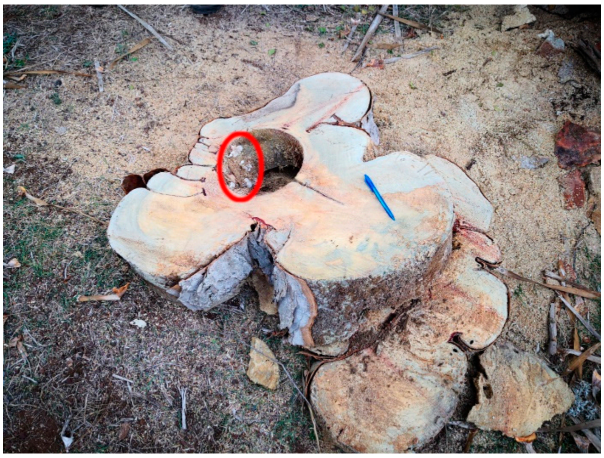

(b)

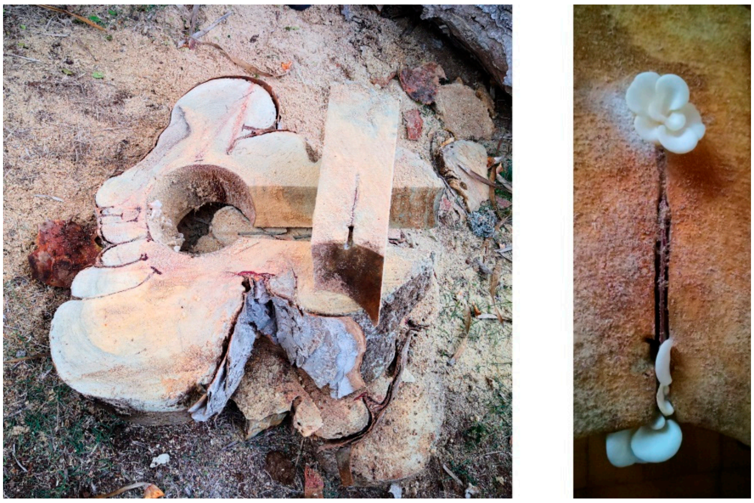

(c)
